# Long-term association between water intake and kidney function in a population at high cardiovascular risk

**DOI:** 10.1016/j.jnha.2024.100327

**Published:** 2024-08-12

**Authors:** Indira Paz-Graniel, Cristina Valle-Hita, Nancy Babio, Lluís Serra-Majem, Jesus Vioque, María Dolores Zomeño, Dolores Corella, Xavier Pintó, Naomi Cano-Ibáñez, Josep A. Tur, Esther Cuadrado-Soto, J.A. Martínez, Andrés Díaz-López, Laura Torres-Collado, Albert Goday, Rebeca Fernández-Carrión, Mariela Nissenshon, Antoni Riera-Mestre, Eva Garrido-Garrido, Cristina Bouzas, Itziar Abete, Lidia Daimiel, Isabel Cornejo-Pareja, Zenaida Vázquez-Ruiz, Nadine Khoury, Karla Alejandra Pérez-Vega, Jordi Salas-Salvadó

**Affiliations:** a Consorcio Centro de Investigación Biomédica en Red, M.P. Fisiopatología de la Obesidad y Nutrición, Instituto de Salud Carlos III, Madrid, Spain; b Universitat Rovira i Virgili, Departament de Bioquímica i Biotecnología, Alimentaciò, Nutrició, Desenvolupament i Salut Mental (ANUT-DSM), Reus, Spain; c Institut d’Investigació Sanitària Pere Virgili (IISPV), Reus, Spain; d Research Institute of Biomedical and Health Sciences (IUIBS), University of Las Palmas de Gran Canaria & Centro Hospitalario Universitario Insular Materno Infantil (CHUIMI), Canarian Health Service, Las Palmas de Gran Canaria, Spain; e CIBER de Epidemiología y Salud Pública (CIBERESP), Instituto de Salud Carlos III, Madrid, Spain; f Instituto de Investigación Sanitaria y Biomédica de Alicante, Universidad Miguel Hernández (ISABIAL-UMH), Alicante, Spain; g Cardiovascular Risk and Nutrition, Hospital del Mar Medical Research Institute, Barcelona, Spain; h School of Health Sciences, Blanquerna-Ramon Llull University, Barcelona, Spain; i Departament of Preventive Medicine and Public Health, School of Medicine, Valencia, Spain; j Internal Medicine Department. Hospital Universitari Bellvitge, Barcelona, Spain; k Bellvitge Biomedical Research Institute (IDIBELL), Barcelona, Spain; l Clinical Sciences Department, Faculty of Medicine and Health Sciences, Universitat de Barcelona, Barcelona, Spain; m Department of Preventive Medicine and Public Health, University of Granada, Granada, Spain; n Instituto de Investigación Biosanitaria (ibs. GRANADA), Complejo Hospitales Universitarios de Granada/Universidad de Granada, 18071, Granada, Spain; o Research Group on Community Nutrition and Oxidative Stress, University of Balearic Islands, Palma de Mallorca, Spain; p Health Research Institute of the Balearic Islands (IdISBa), Palma de Mallorca, Spain; q Nutritional Control of the Epigenome Group, Precision Nutrition and Obesity Program, IMDEA Food, CEI UAM+CSIC, Madrid, Spain; r Grupo de Investigación VALORNUT-UCM, Departamento de Nutrición y Ciencia de los Alimentos, Facultad de Farmacia, Universidad Complutense de Madrid, 28040 Madrid, Spain; s Department of Nutrition, Food Sciences, and Physiology, Center for Nutrition Research, University of Navarra, Pamplona, Spain; t Precision Nutrition and Cardiometabolic Health Program, IEA Food, CEI UAM+CSIC, Madrid, Spain; u Departament of Medicine and Endocrinology, University of Valladolid, Spain; v Nutrition and Mental Health (NUTRISAM) Research Group, Nutrition and Public Health Unit, Universitat Rovira I Virgili, Reus 43204, Spain; w IMIM, Endocrinology and Diabetes Unit, Hospital del Mar, Barcelona, Spain; x Departament de Medicina, Universitat Autónoma de Barcelona, Spain; y Primary Care Center Zaidín-Center, Andalusian Health Service, Granada, Spain; z Departamento de Ciencias Farmacéuticas y de la Salud, Faculty de Farmacia, Universidad San Pablo-CEU, CEU Universities, Boadilla del Monte, Spain; A Virgen de la Victoria Hospital, Department of Endocrinology, Instituto de Investigación Biomédica de Málaga (IBIMA), University of Málaga, 29010 Málaga, Spain; B Department of Preventive Medicine and Public Health, Instituto de Investigación Sanitaria de Navarra (IdiSNA), University of Navarra, 31008 Pamplona, Spain

**Keywords:** Plain water, Tap water, Kidney function, Glomerular filtration rate, Elderly, PREDIMED-Plus study

## Abstract

**Objectives:**

The evidence on water intake in the prevention of kidney function decline is scarce at population level in well-being individuals at high cardiovascular risk. Therefore, we aimed to longitudinally evaluate the associations between total water intake and subtypes and kidney function, through estimated-Glomerular Filtration Rate (eGFR).

**Methods:**

Three-year prospective analysis conducted in 1986 older adults (aged 55–75 year) with overweight/obesity and metabolic syndrome from the PREDIMED-Plus study. Water intake was assessed using validated beverage and food frequency questionnaires. Serum creatinine-based eGFR (SCr-based eGFR; ml/min/1.73 m^2^) was estimated using the CKD-EPI equation at baseline, one-year and 3-years of follow-up. Mixed-effects linear regression models were fitted to evaluate the associations between baseline total water intake and subtypes, and SCr-based eGFR over 3-years of follow-up.

**Results:**

Participants in the highest baseline tertile of total water intake, plain water and water from all fluids showed a lower decrease in SCr-based eGFR after 3-years of follow-up, compared to those in the lowest tertile. Participants with the highest tap water consumption showed a lower SCr-based eGFR decline after 1-year and 3-years of follow-up, in comparerd to participants in the lowest intake category (T3 vs. T1: β: 1.4 ml/min/1.73 m^2^; 95%CI: 0.5–2.3, β: 1.0; 95%CI: 0.1–2.0, respectively).

**Conclusions:**

Plain water rather than other water sources, and especially tap water, was associated with lower kidney function decline assessed through eGFR over 3-years of follow-up, in older individuals at high cardiovascular risk.

**Trial registration:**

ISRCTN89898870. Retrospectively registered on 24 July 2014

## Highlights

• Water intake might prevent kidney function decline.
• Plain water might preserve kidney function in individuals at high cardiovascular risk.
• Tap water rather than bottled water might be beneficial for kidney health.

## 1 Introduction

Kidney function decline is a common ageing condition, which could be exacerbated by comorbidities such as obesity, diabetes and/or hypertension, and that could lead to the onset of chronic kidney disease (CKD) [1]. CKD is estimated to affects approximately 10% of the worldwide population [2], and poses a considerable disease burden to the health system increasing the risk of cardiovascular events and other complications, hospitalization and/or premature death [3]. Therefore, preserving kidney function is crucial to ensure quality of life and to decrease adverse health outcomes, especially in older individuals with comorbidities [4,5].

To prevent or delay CKD progression, current clinical practice guidelines stress the establishment of lifestyle modifications such as salt restriction, a low-protein diet, avoidance of sugar-sweetened beverages, and regular exercise [6]. However, recommendations for water intake are not usually established mainly because of the limited evidence on the relationship between the frequency and amount of water intake and kidney function. Currently, the evidence is limited to the need of fluids restriction in late stages of CKD [6], but there is not a clear recommendation on the amount and type of water intake needed (total water intake, plain water, water from fluids, water from foods) to prevent CKD in wellbeing and/or at high risk populations.

Some observational studies have evaluated the reno-protective effect of water intake in both, general population and patients with CKD [[7], [8], [9], [10], [11]], but controversial results have been reported [12]. To our knowledge, only one randomized clinical trial of patients at CKD stage 3 has evaluated the effect of water supplementation compared to the usual fluid intake, and no effect was reported on kidney function after 12 months [7]. The inconsistencies among previous studies might reside in methodological aspects such as the formulas used to estimate the glomerular filtration rate (eGFR), water and fluid intake assessment tools utilized, type of population studied and follow-up time. As consequence, it is still unclear whether the amount of total water intake and its sources (plain water, beverages or food moisture-driven) can contribute to the kidney function maintenance. This is an important issue, as water intake from food and beverages can entail the intake of energy and nutrients such as potassium, sodium, and phosphorous, that could also influence renal function or CKD complications.

Due to the scarce and inconsistent evidence in the field, the study of the potential prospective associations between water intake and renal function it is essential in terms of public health, especially for individuals with underlying comorbid conditions such as overweight/obesity and metabolic syndrome (MetS). For this reason, the main objective of the present analysis was to evaluate the association between total water intake and its subtypes with kidney function evaluated through the eGFR, over 3-years of follow-up, in a large cohort of older individuals with MetS. We hypothesize that individuals with higher amount of total water intake will present a lower kidney function decline than those with lower intake.

## 2 Methods

### 2.1 Study design and participants

The current study is a 3-year prospective analysis conducted within the framework of the PREDIMED-Plus (PREvención con DIeta MEDiterránea plus) study, which included individuals with overweight/obesity and metabolic syndrome. A detailed explanation of the PREDIMED-Plus inclusion/exclusion criteria and study design has been extensively described elsewhere [13] (Supplementary Material 1), and the protocol can be accessed at https://www.predimedplus.com. The trial was registered at the International Standard Randomized Controlled Trial registry (https://www.isrctn.com/ISRCTN89898870) on July 2014. All participants provided written informed consent and Research Ethics Committees from each of participating centers approved the final protocol.

For the present prospective study, the main analysis was performed using data from 10 of the 23 recruiting centers participating in the fluid intake assessment sub-study. Participants without baseline SCr (serum creatinine) information and reporting implausible total energy intake (women <500 and >3,500 kcal/d and men <800 and >4,000 kcal/d) [14] were excluded from the analysis. Moreover, data from the LIKIDI sub-project conducted in 5 of the 23 PREDIMED-Plus centers were used for the SCr-CysC (cystatin C) based-eGFR secondary analysis provided.

### 2.2 Water intake assessment

At baseline, beverage intake was recorded by trained dietitians using a validated semi-quantitative 32-item Beverage Intake Assessment Questionnaire (BIAQ) [15]. Daily total fluid intake from beverages was computed as the sum of all beverages consumed. The water and nutrient content in beverages were mostly estimated using the Spanish CESNID and BEDCA Spanish food composition databases [16,17].

For the current analysis, plain water intake (mL/d) was estimated based on tap and bottled water intakes according to responses to the BIAQ. Water from fluids (mL/d) was calculated from the water content in all beverages based on BIAQ responses, except for tap and bottled water. Water intake from all fluids (mL/d) was computed by adding plain water intake plus water from fluids. Water from foods (mL/d) corresponded to existing water in food sources based on responses to a 143-item semi-quantitative validated Food Frequency Questionnaire (FFQ) [18]. Total daily energy and nutrient intake were estimated using two Spanish food composition databases [19,20]. Finally, total water intake encompassed water from all fluids and water from food.

### 2.3 Kidney function measurements

The primary outcome for the present study was SCr based-eGFR over 3-years of follow-up. In addition, CysC-SCr based-eGFR (considered a more accurate biomarker of kidney function [21,22] over 3-years of follow-up was evaluated in a subsample of participants (n = 619) and considered a secondary endpoint. CKD Epidemiology Collaboration equations for Caucasian individuals were used to estimate SCr and CysC-SCr based-eGRF [23]. At baseline, one-year and 3-years of follow-up, blood samples were obtained after an overnight fast. SCr levels were determined by the enzymatic creatinine assay method (coefficient of variation <4%) and CysC concentrations by Siemens Atellica NEPH 630 (Siemens Healthineers, Marburg, Germany) nephelometer using the Atellica CH CYSC_2 (Siemens Healthcare GmbH) assay (limit of quantitation 0.25 mg/L; coefficient of variation <10%).

### 2.4 Assessment of covariates

PREDIMED-Plus staff collected socio-demographic and lifestyle information, including age, sex, educational level, dietary intake, physical activity, smoking, medication use and history of disease. Adherence to an energy-reduced MedDiet was assessed using a validated 17-item questionnaire [23]. Leisure-time physical activity was estimated using the validated Registre Gironí del Cor (REGICOR) questionnaire [24]. Anthropometric variables were measured in duplicate following the trial protocol.

### 2.5 Statistical analysis

For the present study, we used the PREDIMED-Plus database updated until August 2021. Participants were categorized into tertiles based on total water consumption (plain water + water from foods + water from all fluids) at baseline. To evaluate differences among tertiles of total water intake for the baseline characteristics of the study population, one-way ANOVA and chi-square tests were performed, as appropriate. Descriptive data were expressed as means ± SD for continuous variables and percentage (%) and number for categorical variables.

Longitudinal associations (β-coefficients and 95% confidence intervals) between tertiles of types of water consumption at baseline and SCr based-eGFR(ml/min/1.73 m^2^) over 3-years of follow-up were examined using linear mixed-effects models with random intercepts at recruitment center, cluster family and participant level. The first tertile (low intake) was considered the reference category. The models were adjusted for age (years), sex (women/men), visit-time (0, 1 or 3 years), BMI (kg/m^2^), educational level (primaryeducation or lower/secondary education/academic or graduate), smoking habit (never/former/current), total energy intake (kcal/day), physical activity (METsmin/week in tertiles), prevalence of diabetes (yes/no), hypertension (yes/no), renal drugs (including angiotensin-converting enzyme inhibitors and angiotensin II receptor blockers, yes/no), diuretics use (yes/no), energy-reduced MedDiet adherence (points in tertiles), intervention group (intervention/control), dietary intake of total protein (g/day) and sodium (mg/day), and participating center (in quartiles by number of participants), as fixed effect. Models assessing the association between plain water, water from fluids, water from food and water from all fluids with SCr based-eGFR were further adjusted for other sources of water.

We conducted a secondary analysis to assess whether types of water intake were associated with eGFR estimated through the formula which combines CysC and SCr (CysC-SCr based-eGFR) over 3-years of follow-up. Moreover, to test the robustness of the results, the main analyses were repeated by excluding participants with type 2 diabetes (T2D),albuminuria (Urine Albumin/Creatinine Ratio (UACR) ≥300 mg/g) and CKD (defined as abnormalities of kidney function (eGFR < 60 ml/min/1.73 m^2^ or ACR ≥ 30 mg/mmol [in at least 2 occasions -90 days apart-]) or structure present for more than 3 months [25] at baseline. Effect modification by sex (women/men), age categories (<65 y/≥65 y) and intervention group (intervention/control) was tested by including multiplicative interaction terms between these variables and types of water consumed in the linear mixed-effects models. Lastly, we evaluated the associations between tap and bottled water consumption at baseline and SCr based-eGFR over 3-years of follow-up. For each type of plain water, tertiles of consumption were calculated. Models were adjusted by the aforementioned confounders.

Statistical analyses were conducted using Stata/SE software, version 17.0 (StataCorp, College Station, TX) and tests were considered statistically significant at a two-tailed p-value <0.05.

## 3 Results

**Fig. 1:**
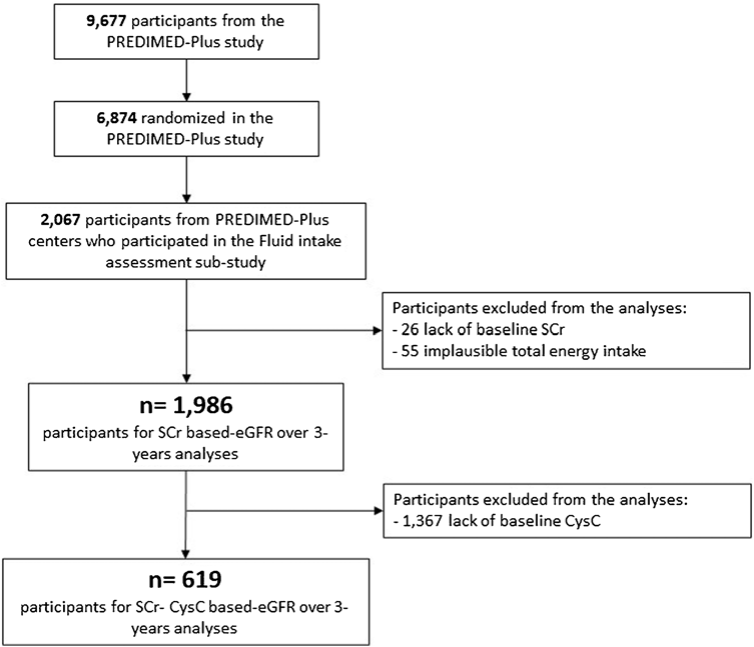
Flowchart of the study participants.

Among 2,067 participants who were available for the fluid intake assessment, 26 individuals with SCr missing data and 55 reporting implausible total energy intake were excluded. Therefore, a total of 1,986 individuals (mean age 64.9 ± 4.9 years and 50.3% women) were included in the SCr based-eGFR analysis. Moreover, 1,367 individuals were excluded from the SCr-CysC based-eGFR analysis due to CysC missing data, remaining a final sample size of 619 participants (Fig. 1).

The general characteristics of the study population according to tertiles of total water intake at baseline are presented in Table 1. Participants presented a baseline mean (±SD) of SCr based-eGFR of 83.1 ± 14.3 ml/min/1.73 m^2^. The mean (±SD) intake of total water, plain water, water from fluids and water from foods, and water from all fluids in mL/d were 2,921 ± 742, 1,040 ± 471, 847 ± 467, 1,033 ± 288, and 1,887 ± 656, respectively. Compared to participants in the lowest tertile of total water intake, those in the highest were more likely to be younger, physically active and use less angiotensin-converting enzyme inhibitors. They also have a higher MedDiet adherence, and dietary intake of total energy, protein, fiber, potassium, sodium, magnesium and phosphorus, and a lower intake of fat.

**Table 1:** Baseline characteristics of the study participants according to tertiles of total water intake in the PREDIMED-Plus (n = 1986).

	Total water intake (mL/d)			
	All population	T1 (lowest)	T2	T3 (highest)
	(n = 1986)	(n = 662)	(n = 662)	(n = 662)
Total water intake, mL/d	2,921 ± 743	2,167 ± 301	2,854 ± 174	3,743 ± 533^a^
Age, years	64.9 ± 4.9	65.3 ± 4.8	64.8 ± 4.8	64.5 ± 5.0^a^
Women, n (%)	50.3 (1,000)	51.5 (341)	51.6 (342)	47.8 (317)
Intervention group, % (n)	49.9 (991)	50.7 (336)	48.3 (320)	50.6 (335)
BMI, kg/m^2^	32.6 ± 3.5	32.5 ± 3.4	32.7 ± 3.5	32.5 ± 3.5
Body weight, kg	86.5 ± 12.9	85.5 ± 12.6	86.9 ± 12.6	87.0 ± 13.6
Waist circumference, cm	107.7 ± 9.8	107.7 ± 10.1	107.9 ± 9.3	107.6 ± 10.1
Smoking status, % (n)				
Never smoked	45.3 (901)	47.5 (315)	45.7 (303)	42.7 (283)
Former smoker	42.2 (838)	39.7 (263)	42.9 (284)	43.9 (291)
Current smoker	12.4 (247)	12.6 (84)	11.3 (75)	13.2 (88)
Education level, % (n)				
Primary education	51.8 (1,029)	5.1 (372)	49.8 (330)	49.4 (327)
Secondary education	27.8 (553)	25.0 (166)	29.7 (197)	28.7 (1909
Academic or graduate	20.3 (404)	18.7 (124)	20.3 (135)	21.9 (145)
Physical activity, METS/min/week	2,459 ± 2,348	2,296 ± 2,268	2,453 ± 2,475	2,627 ± 2,286^a^
Total water intake, mL/d	2,921 ± 743	2,167 ± 301	2,854 ± 174	3,743 ± 533^a^
Plain water, mL/d	1,041 ± 471	704 ± 356	1,030 ± 370	1,386 ± 411^a^
Water from fluids, mL/d	847 ± 467	568 ± 278	807 ± 335	1,165 ± 532^a^
Water from foods, mL/d	1,034 ± 288	894 ± 236	1,016 ± 238	1,190 ± 304^a^
Water from all fluids, mL/d	1,888 ± 657	1,272 ± 341	1,838 ± 272	2,552 ± 529^a^
Dietary assessment				
erMedDiet score, 17-points	8.5 ± 2.5	8.2 ± 2.4	8.3 ± 2.6	8.9 ± 2.6^a^
Energy intake, kcal/d	2,395 540	2,238 ± 541	2,386 ± 502	2,565 ± 527^a^
Protein intake, % energy	16.9 ± 2.7	16.6 ± 2.7	16.9 ± 2.6	17.2 ± 2.8^a^
Fat intake, % energy	39.3 ± 6.3	39.9 ± 6.7	39.6 ± 6.3	38.3 ± 5.9^a^
Carbohydrate intake, % energy	40.9 ± 6.8	40.7 ± 7.2	40.6 ± 6.6	41.3 ± 6.5
Fiber intake, g/day	27.1 ± 8.8	24.0 ± 7.7	26.5 ± 7.7	30.9 ± 9.4^a^
Potassium intake, mg/d	4,616 ± 1,068	4,090 ± 901	4,540 ± 889	5,217 ± 1,087^a^
Calcium intake, mg/d	9.6 ± 0.5	9.6 ± 0.5	9.7 ± 0.5	9.6 ± 0.7
Sodium intake, mg/d	2486 ± 774	2309 ± 769	2476 ± 766	2673 ± 745^a^
Magnesium intake, mg/d	433.7 ± 108.4	388.2 ± 99.3	425.6 ± 93.2	487.2 ± 108.2^a^
Phosphorus intake, mg/d	1811 ± 424	1645 ± 412	1785 ± 374	2003 ± 406^a^
Iron intake, mg/d	16.9 ± 3.9	15.3 ± 3.6	16.8 ± 3.4	18.8 ± 3.8^a^
Creatinine, mg/dl	0.9 ± 0.2	0.9 ± 0.2	0.9 ± 0.2	0.9 ± 0.2
Cystatin C, mg/dl	1.1 ± 0.2	1.1 ± 0.2	1.1 ± 0.2	1.1 ± 0.2
eGFR, ml/min/1.73 m^2^^b^	83.1 ± 14.3	83.2 ± 14.1	82.6 ± 14.8	83.4 ± 14.0
Type 2 diabetes, % (n)	31.5 (626)	33.84 (224)	31.57 (209)	29.15 (193)
Hypertension, % (n)	83.8 (1,664)	85.1 (563)	83.1 (550)	83.2 (551)
Hypercholesterolemia, % (n)	72.4 (1,438)	72.8 (482)	72.5 (480)	71.9 (476)
Medication use, % (n)				
Lipid-lowering drugs	52.0 (1,032)	51.7 (342)	53.3 (353)	50.9 (337)
Oral blood glucose-lowering drugs	26.2 (521)	27.8 (184)	26.6 (176)	24.3 (161)
Insulin treatment	4.3 (86)	3.8 (25)	5.3 (35)	3.9 (26)
Antihypertensive drugs	79.3 (1,575)	79.3 (525)	80.1 (530)	78.6 (520)
ARBs	37.7 (749)	36.4 (241)	38.5 (255)	38.2 (253)
ACEis	29.0 (575)	32.8 (217)	27.5 (182)	26.6 (176)^a^
Diuretics	41.5 (825)	41.1 (272)	42.5 (281)	41.1 (272)

Data expressed as means ± standard deviations for continuous variables and percentage (number) for categorical variables.

ACEis, Angiotensin-Converting Enzyme Inhibitors; ARBs, Angiotensin II receptor blockers; BMI, Body Mass Index; eGFR, Estimated Glomerular Filtration Rate; erMedDiet, energy-restricted Mediterranean diet, METS, Metabolic Equivalent of Task; T, tertile.

a P-value for comparisons between total water intake was calculated was calculated by one-way analysis of variance test or chi-square for continuous and categorical variables, respectively. P-value <0.05.

b eGFR was estimated using EPI-CKD equation based on serum creatinine.

**Fig. 2:**
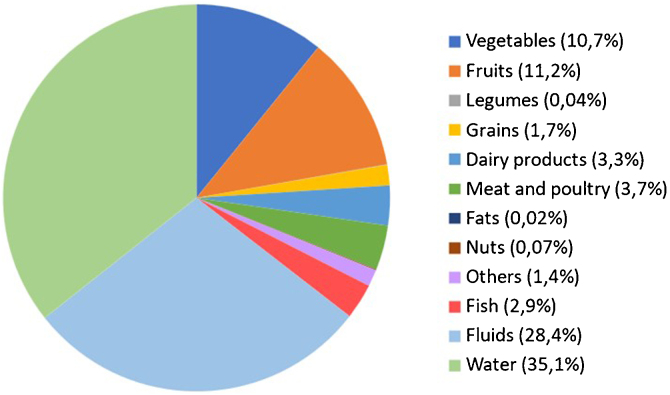
Contribution (%) of food groups and fluids to total water intake. The contribution of fat and nuts to total water intake was lower than 1%. Fluids category includes: natural fruit juices, bottled fruit juices, natural vegetable juices, bottled vegetable juices, whole milk, semi-skimmed milk, skimmed milk, drinking yogurt, milkshakes, vegetable drinks, soups, jellies and sorbets, soda, light/zero soda, espresso, coffee, tea, beer, non-alcoholic beer, wine, spirits, mixed alcoholic drinks, energy drinks, sports drinks and meal replacement shakes.

Fig. 2 depicts the percentage of contribution of different types of fluids and food groups to the total water intake. Plain water and fluids contributed to 35.1% and 28.4% of total water intake, respectively. While food accounted for the remaining, which mainly came from fruit (11.2%) and vegetables (10.7%).

Table 2 summarizes the associations between tertiles of baseline total water and different types of water consumption and SCr-based eGFR over 3-years of follow-up. After controlling for several potential confounders, compared to those in the lowest tertile, participants in the highest baseline tertile intake of total water intake, plain water and water from all fluids showed a lower decrease in with SCr-based eGFR after 3-years of follow-up. This association was also observed at 1-year of follow-up for baseline plain water (β:1.3 ml/min/1.73 m^2^; 95%CI: 0.1–2.4).

**Table 2:** Associations between types of water consumption at baseline and changes in creatinine-based eGFR over 3 years of follow-up (n = 1986).

	Tertiles of water consumption						
	T1 (lowest)	T2	T3 (highest)	T2 vs. T1 difference	p-value	T3 vs. T1 difference	p-value
**Total water intake, mL/d**	**2167 ± 302**	**2854 ± 174**	**3743 ± 533**				
N	662	662	662				
**eGFR (ml/min/1.72 m^2^)**							
Baseline	84.3 (81.5–87.1)	82.7 (79.9–85.5)	82.4 (79.6–85.2)				
1-year	82.9 (80.2–85.8)	81.7 (78.9–84.5)	81.5 (78.7–84.3)				
1-year change	−1.3 (−2.0 to −0.6)	−1.0 (−1.7 to −0.3)	−0.9 (−1.6 to −0.2)	0.3 (−0.7 to 1.4)	0.526	0.4 (−0.6 to 1.4)	0.426
3-years	80.9 (78.1–83.7)	79.9 (77.1–82.7)	80.3 (77.5–83.1)				
3-years change	−3.4 (−4.1 to −2.6)	−2.8 (−3.6 to −2.1)	−2.1 (−2.8 to −1.3)	0.6 (−0.5 to 1.6)	0.291	**1.3 (0.3 to 2.4)**	**0.012**
**Plain water, mL/d**	**702 ± 269**	**1293 ± 46**	**1814 ± 169**				
N	1123	553	310				
**eGFR (ml/min/1.72 m^2^)**							
Baseline	83.8 (81.1–86.5)	82.6 (79.8–85.5)	81.4 (78.4–84.4)				
1-year	82.4 (79.6–85.1)	81.8 (79.0–84.7)	81.3 (78.2–84.7)				
1-year change	−1.4 (−1.9 to −0.9)	−0.8 (−1.6 to −0.0)	−0.2 (−1.2 to 0.9)	0.6 (−0.3 to 1.6)	0.188	**1.3 (0.1 to 2.4)**	**0.039**
3-years	80.6 (77.9–83.4)	79.9 (77.0–82.7)	80.1 (77.1–83.2)				
3-years change	−3.2 (−3.7 to −2.6)	−2.7 (−3.5 to −1.9)	−1.3 (−2.4 to −0.23)	0.4 (−0.6 to 1.4)	0.410	**1.9 (0.6 to 3.1)**	**0.003**
**Water from fluids, mL/d**	**410 ± 160**	**775 ± 92**	**1354 ± 408**				
N	662	662	662				
**eGFR (ml/min/1.72 m^2^)**							
Baseline	83.0 (80.2–85.8)	83.5 (80.7–86.3)	82.9 (80.1–85.7)				
1-year	81.6 (78.8–84.5)	82.9 (80.1–85.7)	81.6 (78.8–84.4)				
1-year change	−1.3 (−2.1 to −0.6)	−0.6 (−1.3 to 0.1)	−1.2 (−2.0 to −0.5)	0.8 (−0.3 to 1.8)	0.153	0.1 (−0.9 to 1.1)	0.854
3-years	79.9 (77.1–82.7)	80.9 (78.0–83.7)	80.3 (77.5–83.1)				
3-years change	−3.1 (−3.8 to −2.3)	−2.6 (−3.4 to −1.9)	−2.6 (−3.3 to −1.8)	0.5 (−0.6 to 1.5)	0.399	0.5 (−0.6 to 1.5)	0.370
**Water from foods, mL/d**	**740 ± 120**	**1006 ± 67**	**1354 ± 201**				
N	662	662	662				
**eGFR (ml/min/1.72 m^2^)**							
Baseline	82.4 (79.6–85.3)	83.2 (80.4–86.1)	83.7 (80.8–86.5)				
1-year	81.8 (79.0–84.7)	81.9 (79.1–84.8)	82.4 (79.5–85.2)				
1-year change	−0.6 (−1.3 to 0.1)	−1.3 (−2.0 to −0.6)	−1.3 (−2.0 to −0.6)	−0.7 (−1.7 to 0.3)	0.174	−0.7 (−1.7 to 0.3)	0.193
3-years	80.2 (77.3–83.0)	80.3 (77.5–83.1)	80.6 (77.8–83.5)				
3-years change	−2.3 (−3.0 to −1.5)	−3.0 (−3.7 to −2.2)	−3.0 (−3.8 to −2.3)	−0.7 (−1.8 to 0.3)	0.185	−0.8 (−1.8 to 0.3)	0.138
**Water from all fluids, mL/d**	**1222 ± 290**	**1832.8 ± 152**	**2608.4 ± 470**				
N	662	662	662				
**eGFR (ml/min/1.72 m^2^)**							
Baseline	84.2 (81.3–87.0)	83.4 (80.6–86.2)	81.8 (78.9–84.6)				
1-year	82.7 (79.9–85.5)	82.4 (79.6–85.3)	81.0 (78.2–83.9)				
1-year change	−1.5 (−2.2 to −0.8)	−1.0 (−1.7 to −0.3)	−0.7 (−1.4 to 0.0)	0.5 (−0.5 to 1.5)	0.347	0.8 (−0.3 to 1.8)	0.144
3-years	80.8 (78.0–83.6)	80.3 (77.5–83.2)	80.0 (77.1–82.8)				
3-years change	−3.3 (−4.1 to −2.6)	−3.1 (−3.8 to −2.4)	−1.8 (−2.5 to −1.1)	0.3 (−0.8 to 1.3)	0.640	**1.5 (0.5 to 2.6)**	**0.004**

Linear Mixed Models (β-coefficients (ml/min/1.73 m^2^) and 95% CI) were used to assess the longitudinal associations between types of water consumption at baseline and changes in eGFR (creatinine) over 3 years of follow-up. Model was adjusted for age, sex, visit-time, body mass index (kg/m^2^), educational level (primary or lower, secondary or academic or graduate), smoking habit (never, former or current), total energy intake (kcal/day), physical activity (METs min/week in tertiles), prevalence of diabetes (yes/no), hypertension (yes/no), renal drugs use (yes/no), diuretics use (yes/no), energy reduced Mediterranean diet adherence (in tertiles), intervention group, dietary intakes of total protein(gr/d) and sodium (mg/d), and participating center (in quartiles by number of participants).

Plain water, water from fluids, water from all fluids, and water from food models were adjusted for other water source.

Abbreviations: eGFR, estimated glomerular filtration rate.

Sensitivity analyses were performed using the eGFR through the CysC-SCr equation (Supplementary Table S1). Although the magnitude of the previous estimated results was markedly reduced and statistical significance has been lost for all types of water consumption, the association persisted in case of plain water after one-year of follow-up. Compared to participants in the lowest tertile of plain water, those in the highest presented an increase in CysC-SCr based-eGFR after one-year of follow-up (β:2.8 ml/min/1.73 m^2^; 95%CI: 0.7–4.8). When the main analysis was repeated by excluding participants with baseline T2D, albuminuria (UACR ≥300 mg/g) and CKD, the results did not substantially change, except for water from foods, which showed a negative association with SCr based-eGFR over 3-years of follow-up (At 1 year: T2 vs. T1: β: −1.4 ml/min/1.73 m^2^; 95%CI: −2.6 to −0.2; T3 vs. T1: β: −1.4 ml/min/1.73 m^2^; 95%CI: −2.6 to −0.3. At 3-years: T2 vs. T1: β: −1.3 ml/min/1.73 m^2^; 95%CI: −2.6 to −0.1; T3 vs. T1: β: −1.2 ml/min/1.73 m^2^; 95%CI: −2.4−0.1; Supplementary Table S2) in individuals without baseline T2D (n = 1,360). No significant interactions for sex, age, intervention group and types of water consumption were shown, except for intervention group and total water intake (p = 0.011) or water from all fluids (p = 0.018). When analyses were repeated by intervention group, the associations between total water intake and eGFR over 3-years of follow-up remained significant and in the same direction for both groups. Findings for water from all fluids by intervention group, showed the same trend although the significance was lost for eGFR over 3-years of follow-up in the intervention group (Supplementary Table S3). Whether the type of plain water consumed (tap or bottled) was associated with SCr-based eGFR over 3-years of follow-up was also examined (Table 3). The amount of baseline tap water consumed was associated with SCr-based eGFR over 3-years. Participants in the highest baseline tertile of tap water present a lower decrease in SCr-based eGFR after one-year (T2 vs. T1: β: 1.6 ml/min/1.73 m^2^; 95%CI: 0.3−2.9. T3 vs. T1: β: 1.4 ml/min/1.73 m^2^; 95%CI: 0.5–2.3) and 3-years of follow-up (T2 vs. T1: β: 1.8 ml/min/1.73 m^2^; 95%CI: 0.4−3.1. T3 vs. T1: β: 1.0; 95%CI: 0.1–2.0), compared to those in the lowest tertile. Compared to participants in the lowest tertile of bottled water intake, those in the second tertile presented a higher decline in SCr-based eGFR. However, this association was attenuated when comparing those participants in the highest tertile with those in the lowest tertile.

**Table 3:** Associations between tap and bottled water at baseline and changes in creatinine-based eGFR over 3 years of follow-up (n = 1986).

	Tertiles of water consumption						
	T1 (lowest)	T2 (lowest)	T3 (highest)	T2 vs. T1 difference	p-value	T3 vs. T1 difference	p-value
**Tap water, mL/d**	0 **±** 0	380 **±** 74	1214 **±** 339				
N	1072	257	657				
**eGFR (ml/min/1.72 m^2^)**							
Baseline	83.5 (80.7–86.2)	83.0 (80.0–86.1)	82.4 (79.5–85.3)				
1-year	81.8 (79.0–84.5)	82.9 (79.8–85.9)	82.0 (79.2–84.9)				
1-year change	−1.7 (2.3 to −1.2)	−0.1 (−1.3 to 1.0)	−0.3 (−1.0 to 0.4)	**1.6 (0.3 to 2.9)**	**0.016**	**1.4 (0.5 to 2.3)**	**0.003**
3-years	80.1 (77.4–82.9)	81.5 (78.4–84.6)	80.1 (77.2–83.0)				
3-years change	−3.3 (−3.9 to −2.8)	−1.5 (−2.7 to −0.3)	−2.3 (−3.0 to −1.5)	**1.8 (0.4 to 3.1)**	**0.008**	**1.0 (0.1 to 2.0)**	**0.025**
**Bottled water, mL/d**	0 **±** 0	673 **±** 28	1469 **±** 237				
N	777	761	448				
**eGFR (ml/min/1.72 m^2^)**							
Baseline	83.3 (80.5–86.2)	83.4 (80.7–86.1)	81.9 (79.0–84.8)				
1-year	83.0 (80.2–85.9)	81.4 (78.6–84.1)	81.2 (78.3–84.1)				
1-year change	−0.3 (−0.9 to 0.3)	−2.0 (−2.7 to −1.4)	−0.7 (−1.6 to 0.2)	**−1.7 (**−**2.7 to** −**0.7)**	**<0.001**	−0.4 (−1.5 to 0.7)	0.514
3-years	81.3 (78.5–84.1)	79.6 (77.0–82.5)	79.5 (76.6–82.4)				
3-years change	−2.0 (−2.7 to −1.4)	−3.6 (−4.3 to −2.9)	−2.5 (−.3.4 to 1.5)	−**1.6 (**−**2.6 to** −**0.6)**	**0.001**	−0.4 (−1.5 to 0.7)	0.480

Linear Mixed Models (β-coefficients (ml/min/1.73m^2^) and 95% CI) were used to assess the longitudinal associations between types of water consumption at baseline and changes in eGFR (creatinine + cystatin) over 3 years of follow-up. Model was adjusted for age, sex, visit-time, body mass index (kg/m^2^), educational level (primary or lower, secondary or academic or graduate), smoking habit (never, former or current), total energy intake (kcal/day), physical activity (METs min/week in tertiles), prevalence of diabetes (yes/no), hypertension (yes/no), renal drugs use (yes/no), diuretics use (yes/no), energy reduced Mediterranean diet adherence (in tertiles), intervention group, dietary intakes of total protein(gr/d) and sodium (mg/d), and participating center (in quartiles by number of participants).

Plain water, water from fluids, water from all fluids, and water from food models were adjusted for other water source.

Abbreviations: eGFR, estimated glomerular filtration rate.

## 4 Discussion

To our knowledge, this is the first prospective study analyzing the associations between the amount of different water intake sources and kidney function (using SCr and CysC biomarkers to estimate eGFR) in a population at cardiometabolic risk using a validated specific fluid intake questionnaire. We found that a baseline higher daily total water intake, plain water and water from all fluids was associated with a lower 3-year kidney decline.

Our findings are consistent with those from some previous population-based studies reporting that higher total water intake is associated with a lower prevalence of CKD and lower eGFR decline [8,12,26]. High-to-moderate intake of water was cross-sectionally associated with 19% lower odds of renal impairment (≤60 mL/min/1.73 m^2^) compared to low total water intake in adults [8]. Similarly, in an Italian adult population, baseline water intake was cross-sectionally related to kidney function [26]. By contrast, in a cross-sectional analysis of a US population with CKD stage-III, low daily plain water intake was associated with higher odds of CKD in comparison to high intake, but no association was observed for total water intake [12]. In contrast, in individuals with CKD [9], total and plain water intake showed a U-shaped association with eGFR after a 2.7-years of follow-up. While, in the only randomized clinical trial analyzing the effect of water supplementation in individuals with CKD, no effect was reported after 1-year [7]. In our study, no significant associations were observed between different sources of water intake and eGFR in individuals with CKD (n = 97), probably due to the small size and loss of statistical power due to small size. In the present study, the amount of plain water was associated with a lower decrease in creatinine-based eGFR over 3-years of follow-up, and in creatinine-cystatin C-based eGFR at 1- year of follow-up. Discrepancies might lie to the contribution of plain water, other fluids/beverages and food to total water intake. In our population adhering to a MedDiet, food accounted for 37% of total water intake which is much higher than the reported in the NHANES survey [7], partially explaining these differences [27]. In addition, the results from sensitivity analyses by T2D status showing an inverse association between water from food and eGFR in individuals free of T2D back up the relevance of foods and dietary patterns in terms of water sources and kidney health, as non-diabetic individuals showed lower consumption of fruit, legumes, fish and dairy products than people with T2D at baseline (data not shown), that in may impact kidney function in the long term. In case of individuals with diabetes this association was not observed probably because their water food sources were healthier at baseline, probably as a result of being aware their increased risk of disease (reverse causality). The lack of association can also be partially explained due to the small number of diabetic participants compared to those without diabetes (lack of statistical power). Further, the evidence suggests that high intake of total water may be helpful to prevent eGFR decline in older adults with MetS, but not in patients with moderate or advanced CKD in which increased intake of plain water might rise urine flow and potentially accelerate the disease progression [28]. Further, our findings suggest that the type of plain water is important in terms of kidney health, as tap water was associated with lower creatinine-based eGFR decline, while higher intakes of bottled water were not associated with kidney function over 3-years of follow-up. Whether the high content of sodium, calcium, and magnesium of some bottled water brands may be the responsible or not of these associations remains to be explored in the future [29].

Studies exploring the relationship between daily fluid intake (tea, coffee, milk, juices, sweetened-drinks and alcohol) with kidney function were scarce. In a cross-sectional analysis of older Australian people, an inverse association between the amount of fluid intake and the prevalence of CKD was reported [30]. Meanwhile, water intake from food and beverages was not associated with long-term kidney function after a median of 13.1 years of follow-up [31]. Similarly, we did not observe associations between water from fluids and changes in creatinine-based eGFR over 3-years of follow-up. This lack of association might be explained by the fact that, unlike plain water, beverages contain nutrients and compounds such as carbohydrates (including free and added sugar), proteins, sodium, and phosphate additives. Some of them may induce weight gain or an increase in blood glucose and/or urate concentrations, leading to insulin resistance, chronic inflammation and hypertension, all of which have been recognized as CKD risk factors [32,33].

Some studies have explored the associations between total water intake and kidney function activity mediators, such as arginine vasopressin (AVP), providing insights into the potential biological mechanisms behind our results [[34], [35], [36], [37], [38]]. Water intake reduces AVP plasma levels [34], which induce increases in renal plasma flow and glomerular hyperfiltration [39], that have been associated with increased urinary albumin excretion and microalbuminuria [35,36]. Both are recognized markers of CKD and strongly correlated with lower levels of eGFR [7]. It has also been suggested that long-term fluid restriction potentially stimulates the release of AVP, which in addition to increase glomerular filtration [39] might also lower sodium excretion [37], causing chronic kidney damage [36,40] and/or hypertension. Results from animal models with CKD support this hypothesis [38].

Our results should be interpreted with caution. First, our study population was composed of older individuals with MetS which limits the applicability of our findings to younger and healthy populations. Second, due to the observational design of our study, causality cannot be established. Third, individuals included in the current study are under a lifestyle intervention program which may influence our results [41]; nevertheless, our analyses were adjusted by treatment group to control for the potential intervention effect, and results were similar in sensitivity analyses by intervention group. Finally, as individuals in the highest category of water intake showed to have a healthier lifestyle than those in lower categories, we cannot discard residual confounding due to behavioral patterns rather than water intake. Regardless, our study has different strengths: (a) its prospective design, which reduces the possibility of reverse causation bias, (b) the use of a specific validated fluid-intake questionnaire to assess beverage consumption, (c) its large sample size studied and adjustment for several potential confounding factors.

## 5 Conclusions

The results of this research based on a detailed assessment of water intake suggest that in older Spanish individuals at high cardiovascular risk, the amount of plain water, especially tap water, is inversely associated with kidney function decline in the long-term. Although further studies are warranted, our results suggest that plain water intake rather than other water sources is associated with lower kidney function decline. Recommendations to drink water, then, should be part of dietary advice on kidney function preservation and CKD prevention although the results of our study may be replicated with other cohorts and study designs.

## Ethical standards

All participants provided their written informed consent. The study protocol and procedures were approved in accordance with the ethical standards of the Declaration of Helsinki.

## Funding

This work was supported by the official Spanish Institutions for funding scientific biomedical research, CIBER Fisiopatología de la Obesidad y Nutrición (CIBEROBN) and Instituto de Salud Carlos III (ISCIII), through the Fondo de Investigación para la Salud (FIS), which is co-funded by the European Regional Development Fund (six coordinated FIS projects leaded by JS-S and JVi, including the following projects: PI13/00673, PI13/00492, PI13/00272, PI13/01123, PI13/00462, PI13/00233, PI13/02184, PI13/00728, PI13/01090, PI13/01056, PI14/01722, PI14/00636, PI14/00618, PI14/00696, PI14/01206, PI14/01919, PI14/00853, PI14/01374, PI14/00972, PI14/00728, PI14/01471, PI16/00473, PI16/00662, PI16/01873, PI16/01094, PI16/00501, PI16/00533, PI16/00381, PI16/00366, PI16/01522, PI16/01120, PI17/00764, PI17/01183, PI17/00855, PI17/01347, PI17/00525, PI17/01827, PI17/00532, PI17/00215, PI17/01441, PI17/00508, PI17/01732, PI17/00926, PI19/00957, PI19/00386, PI19/00309, PI19/01032, PI19/00576, PI19/00017, PI19/01226, PI19/00781, PI19/01560, PI19/01332, PI20/01802, PI20/00138, PI20/01532, PI20/00456, PI20/00339, PI20/00557, PI20/00886, PI20/01158); the Especial Action Project entitled: Implementación y evaluación de una intervención intensiva sobre la actividad física Cohorte PREDIMED-Plus grant to JS-S; the European Research Council (Advanced Research Grant 2014–2019; agreement #340918) granted to MÁM-G.; the Recercaixa (number 2013ACUP00194) grant to JS-S; grants from the Consejería de Salud de la Junta de Andalucía (PI0458/2013, PS0358/2016, PI0137/2018); the PROMETEO/2017/017 and PROMETEO/2021/21 grants from the Generalitat Valenciana; the SEMERGEN grant, and funds from the 10.13039/501100008530European Regional Development Fund (CB06/03). Study resulting from the SLT006/17/00246 grant, funded by the Department of Health of the Generalitat de Catalunya by the call “Acció instrumental de programes de recerca orientats en l'àmbit de la recerca i la innovació en salut”. We thank the CERCA Programme/Generalitat de Catalunya for institutional support. We thank the Fundación Francisco Soria Melguizo for the financial support. Jordi Salas-Salvadó, is partially supported by 10.13039/501100003741ICREA under the ICREA Academia program. None of the funding sources took part in the design, collection, analysis, interpretation of the data, or writing the report, or in the decision to submit the manuscript for publication. C.V-H. receives a predoctoral grant from the 10.13039/501100002809Generalitat de Catalunya (2022 FI_B100108). NK was funded by a research grant from the Agència de Gestió d’Ajuts Universitaris de Recerca (AGAUR FI, record number: 2021FI_B 00145). EC-S financial support from the Juan de la Cierva Program Training Grant (FJC2020-045377-I, MCIN/AEI/10.13039/501100011033) and by European Union NextGenerationEU/PRTR.

## Author contributions

Study concept and design: I.P-G, N.B and J.S-S. Statistical analyses: I.P-G, and C.V-H. Drafting the manuscript: I.P-G, C.V-H, N.B, and J.S-S. All authors reviewed the manuscript for important intellectual content and approved the final version to be published.

## Conflict of interest

The authors declare no conflict of interest.

## Availability of data and materials

The datasets generated and analyzed during the current study are not expected to be made available outside the core research group, as neither participants’ consent forms nor ethics approval included permission for open access. However, the researchers will follow a controlled data sharing collaboration model, as in the informed consent participants agreed with a controlled collaboration with other investigators for research related to the project’s aims. Therefore, investigators who are interested in this study can contact the PREDIMED Plus Steering Committee by sending a request letter to predimed_plus_scommittee@googlegroups.com. A data sharing agreement indicating the characteristics of the collaboration and data management will be completed for the proposals that are approved by the Steering Committee.
