# Supplementary material for: Long-term association between water intake and kidney function in a population at high cardiovascular risk
Source: J Nutr Health Aging. 2024 Aug 12;28(9):100327. doi: 10.1016/j.jnha.2024.100327 (PMC12880064; doi:10.1016/j.jnha.2024.100327)
Supplement: Supplementary file 1 [file mmc1.docx]

**Supplementary Material**

- Supplementary Material 1. PREDIMED-PLUS trial. Study design
- Supplemental Table 1. Associations between types of water consumption at baseline and changes in creatinine-cystatin C-based eGFR over 3 years of follow-up (n=619).
- Supplemental Table 2. Association between water from foods at baseline and changes in creatinine-based eGFR in individuals without baseline T2D over 3 years of follow-up (n=1,360).
- Supplemental Table 3. Associations between total water intake and total water from all fluids at baseline and changes in creatinine-based eGFR over 3 years of follow-up by intervention group.
- Supplementary Material 1. PREDIMED-PLUS trial. Study design

The PREDIMED-Plus is an ongoing, large, parallel-group, 8-year multicenter, randomized and controlled clinical trial aiming to evaluate the effect of an intensive lifestyle weight loss intervention (based on energy-reduced traditional Mediterranean diet (MedDiet), physical activity promotion and behavioral support) on cardiovascular disease (CVD) morbidity and mortality compared to usual care advice and *ad libitum* MedDiet recommendations. Between October 2013 and December 2016, 6,874 men (55–75 years) and women (60–75 years) with overweight or obesity (BMI ≥27 kg/m^2^ and <40 kg/m^2^) and free from cardiovascular disease at baseline who satisfied at least 3 criteria for the MetS definition^13^ were enrolled by 23 centers working in collaboration with primary care clinics across Spain. Eligible participants were randomly allocated in a 1:1 ratio to the intervention or control group, and couples who share the same household were randomized together as a unit of randomization.

| **Supplemental Table 1.**  **Associations between types of water consumption at baseline and changes in creatinine-cystatin C-based eGFR over 3 years of follow-up (n=619).** | | | | | | | |
| --- | --- | --- | --- | --- | --- | --- | --- |
|  | **Tertiles of water consumption** | | | | | | |
|  | **T1 (lowest)** | **T2** | **T3 (highest)** | **T2 vs. T1 difference** | **p-value** | **T3 vs. T1 difference** | **p-value** |
| **Total water intake, mL/d** | **2137 ± 281** | **2787 ± 170** | **3575 ± 454** |  |  |  |  |
| N | 207 | 206 | 206 |  |  |  |  |
| **eGFR (ml/min/1.72m^2^)** |  |  |  |  |  |  |  |
| Baseline | 79.0 (77.2 to 80.8) | 77.3 (75.5 to 79.0) | 76.0 (74.2 to 77.8) |  |  |  |  |
| 1-year | 78.5 (76.6 to 80.4) | 77.7 (75.9 to 79.5) | 76.2 (74.3 to 78.0) |  |  |  |  |
| 1-year change | -0.5 (-1.7 to 0.7) | 0.4 (-0.8 to 1.6) | 0.1 (-1.1 to 1.3) | 0.9 (-0.8 to 2.7) | 0.282 | 0.6 (-1.1 to 2.3) | 0.467 |
| 3-year | 75.7 (73.8 to 77.6) | 74.6 (72.7 to 76.4) | 73.1 (71.2 to 75.0) |  |  |  |  |
| 3-year change | -3.3 (-4.6 to -2.0) | -2.7 (-3.9 to -1.5) | -2.9 (-4.2 to -1.7) | 0.6 (-1.2 to 2.4) | 0.515 | 0.4 (-1.4 to 2.1) | 0.692 |
| **Plain water, mL/d** | **699 ± 254** | **1300 ±** | **1809 ± 83** |  |  |  |  |
| N | 366 | 162 | 91 |  |  |  |  |
| **eGFR (ml/min/1.72m^2^)** |  |  |  |  |  |  |  |
| Baseline | 77.9 (76.6 to 79.2) | 77.6 (75.6 to 79.6) | 75.3 (72.5 to 78.0) |  |  |  |  |
| 1-year | 77.3 (75.9 to 78.7) | 77.8 (75.8 to 79.9) | 77.4 (74.6 to 80.2) |  |  |  |  |
| 1-year change | -0.6 (-1.5 to 0.3) | 0.3 (-1.1 to 1.6) | 2.1 (0.3 to 3.9) | 0.9 (-0.7 to 2.5) | 0.281 | **2.8 (0.7 to 4.8)** | **0.007** |
| 3-year | 74.3 (72.9 to 75.7) | 75.4 (73.3 to 77.5) | 73.5 (70.6 to 76.3) |  |  |  |  |
| 3-year change | -3.7 (-4.6 to -2.7) | -2.2 (-3.6 to -0.8) | -1.8 (-3.6 to 0.1) | 1.5 (-0.2 to 3.2) | 0.081 | 1.9 (-0.2 to 3.9) | 0.078 |
| **Water from fluids, mL/d** | **426 ± 162** | **775 ± 87** | **1291 ± 322** |  |  |  |  |
| N | 207 | 206 | 206 |  |  |  |  |
| **eGFR (ml/min/1.72m^2^)** |  |  |  |  |  |  |  |
| Baseline | 78.5 (76.7 to 80.3) | 77.2 (75.4 to 79.0) | 76.5 (74.7 to 78.3) |  |  |  |  |
| 1-year | 78.89 (77.0 to 80.7) | 77.5 (75.7 to 79.3) | 76.0 (74.1 to 77.8) |  |  |  |  |
| 1-year change | 0.4 (-0.9 to 1.6) | 0.3 (-0.9 to 1.5) | -0.5 (-1.8 to 0.7) | -0.1 (-1.8 to 1.6) | 0.939 | -0.9 (-2.6 to 0.8) | 0.314 |
| 3-years | 75.7 (73.9 to 77.6) | 74.4 (72.5 to 76.2) | 73.2 (71.3 to 75.1) |  |  |  |  |
| 3-years change | -2.8 (-4.1 to -1.6) | -2.8 (-4.1 to -1.6) | -3.3 (-4.6 to -2.1) | -0.02 (-1.8 to 1.8) | 0.984 | -0.5 (-2.3 to 1.2) | 0.564 |
| **Water from foods, mL/d** | **729 ± 101** | **958 ± 57** | **1260 ± 196** |  |  |  |  |
| N | 207 | 206 | 206 |  |  |  |  |
| **eGFR (ml/min/1.72m^2^)** |  |  |  |  |  |  |  |
| Baseline | 76.6 (74.6 to 78.5) | 78.2 (76.4 to 80.0) | 77.5 (75.5 to 79.4) |  |  |  |  |
| 1-year | 76.1 (74.1 to 78.1) | 78.8 (77.0 to 80.6) | 77.5 (75.5 to 79.4) |  |  |  |  |
| 1-year change | -0.5 (-1.7 to 0.7) | 0.6 (-0.6 to 1.8) | 0.01 (-1.2 to 1.2) | 1.1 (-0.6 to 2.8) | 0.210 | 0.5 (-1.2 to 2.2) | 0.565 |
| 3-years | 73.6 (71.6 to 75.6) | 75.6 (73.8 to 77.4) | 74.1 (72.1 to 76.1) |  |  |  |  |
| 3-years change | -3.0 (-4.2 to -1.7) | -2.6 (-3.9 to -1.4) | -3.4 (-4.6 to -2.1) | 0.4 (-1.4 to 2.1) | 0.701 | -0.4 (-2.2 to 1.4) | 0.672 |
| **Water from all fluids, mL/d** | **1224 ± 251** | **1804 ± 150** | **2524 ± 360** |  |  |  |  |
| N | 207 | 206 | 206 |  |  |  |  |
| **eGFR (ml/min/1.72m^2^)** |  |  |  |  |  |  |  |
| Baseline | 79.7 (77.9 to 81.5) | 76.8 (75.1 to 78.6) | 75.7 (73.9 to 77.5) |  |  |  |  |
| 1-year | 78.9 (77.1 to 80.8) | 77.4 (75.5 to 79.2) | 76.1 (74.2 to 77.9) |  |  |  |  |
| 1-year change | -0.8 (-2.0 to 0.5) | 0.5 (-0.7 to 1.7) | 0.3 (-0.9 to 1.5) | 1.3 (-0.5 to 3.0) | 0.147 | 1.1 (-0.6 to 2.8) | 0.218 |
| 3-years | 76.1 (74.2 to 78.0) | 73.8 (72.0 to 75.6) | 73.5 (71.6 to 75.3) |  |  |  |  |
| 3-years change | -3.6 (-4.9 to -2.3) | -3.1 (-4.3 to -1.8) | -2.3 (-3.5 to -1.1) | 0.6 (-1.2 to 2.3) | 0.540 | 1.3 (-0.5 to 3.1) | 0.143 |
| Linear Mixed Models (β-coefficients (ml/min/1.73m2) and 95% CI) were used to assess the longitudinal associations between types of water consumption at baseline and changes in eGFR (creatinine + cystatin) over 3 years of follow-up. Model was adjusted for age, sex, visit-time, body mass index (kg/m^2^), educational level (primary or lower, secondary or academic or graduate), smoking habit (never, former or current), total energy intake (kcal/day), physical activity (METs min/week in tertiles), prevalence of diabetes (yes/no), hypertension (yes/no), renal drugs use (yes/no), diuretics use (yes/no), energy reduced Mediterranean diet adherence (in tertiles), intervention group, dietary intakes of total protein(gr/d) and sodium (mg/d), and participating center (in quartiles by number of participants).  Plain water, water from fluids, water from all fluids, and water from food models were adjusted for other water source.  Abbreviations: eGFR, estimated glomerular filtration rate. | | | | | | | |

| **Supplemental Table 2. Association between water from foods at baseline and changes in creatinine-based eGFR in individuals without baseline T2D over 3 years of follow-up (n=1,360).** | | | | | | | |
| --- | --- | --- | --- | --- | --- | --- | --- |
|  | **Tertiles of water consumption** | | | | | | |
|  | **T1 (lowest)** | **T2** | **T3 (highest)** | **T2 vs. T1 difference** | **p-value** | **T3 vs. T1 difference** | **p-value** |
| **Water from foods, mL/d** | **739 121** | **1008 66** | **1359 203** |  |  |  |  |
| N | 454 | 453 | 453 |  |  |  |  |
| **eGFR (ml/min/1.72m^2^)** |  |  |  |  |  |  |  |
| Baseline | 82.4 (79.4 to 85.3) | 83.8 (80.9 to 86.7) | 83.5 (80.5 to 86.4) |  |  |  |  |
| 1-year | 82.8 (79.8 to 85.8) | 82.9 (79.9 to 85.8) | 82.6 (79.6 to 85.5) |  |  |  |  |
| 1-year change | 0.48 (-0.35 to 1.31) | -0.88 (-1.72 to -0.05) | -0.93 (-1.76 to -0.09) | **-1.4 (-2.6 to -0.2)** | **0.02** | **-1.4 (-2.6 to -0.3)** | **0.01** |
| 3-years | 81.0 (78.0 to 83.9) | 81.0 (78.1 to 84.0) | 80.9 (77.9 to 83.9) |  |  |  |  |
| 3-years change | -1.38 (-2.24 to -0.52) | -2.71 (-3.58 to -1.85) | -2.58 (-3.44 to -1.73) | **-1.3 (-2.6 to -0.1)** | **0.03** | -1.2 (-2.4 to 0.1) | 0.05 |
| Linear Mixed Models (β-coefficients (ml/min/1.73m2) and 95% CI) were used to assess the longitudinal associations between types of water consumption at baseline and changes in eGFR (creatinine + cystatin) over 3 years of follow-up. Model was adjusted for age, sex, visit-time, body mass index (kg/m^2^), educational level (primary or lower, secondary or academic or graduate), smoking habit (never, former or current), total energy intake (kcal/day), physical activity (METs min/week in tertiles), hypertension (yes/no), renal drugs use (yes/no), diuretics use (yes/no), energy reduced Mediterranean diet adherence (in tertiles), intervention group, dietary intakes of total protein(gr/d) and sodium (mg/d), and participating center (in quartiles by number of participants).  Water from food models was adjusted for other water source.  Abbreviations: eGFR, estimated glomerular filtration rate. | | | | | | | |

| **Supplemental Table 3.** Associations between total water intake and total water from all fluids at baseline and changes in creatinine-based eGFR over 3 years of follow-up by intervention group. | | | | | | | |
| --- | --- | --- | --- | --- | --- | --- | --- |
| **Intervention group (n = 909)** | | | | | | | |
| **Tertiles of water consumption** | | | | | | | |
|  | **T1 (lowest)** | **T2** | **T3 (highest)** | **T2 vs. T1 difference** | **p-value** | **T3 vs. T1 difference** | **p-value** |
| **Total water intake, mL/d** | **2145 ± 272** | **2787 ± 160** | **3521 ± 334** |  |  |  |  |
| N | 303 | 303 | 303 |  |  |  |  |
| **eGFR (ml/min/1.72m^2^)** |  |  |  |  |  |  |  |
| Baseline | 83.3 (79.9 to 86.7) | 82.6 (79.2 to 85.9) | 83.5 (80.1 to 86.9) |  |  |  |  |
| 1-year | 82.1 (78.7 to 85.5) | 82.9 (79.6 to 86.4) | 83.2 (79.7 to 86.6) |  |  |  |  |
| 1-year change | -1.30 (-2.41 to -0.20) | 0.36 (-0.72 to 1.44) | -0.00 (-1.07 to 1.07) | **1.67 (0.12 to 3.22)** | **0.03** | 1.30 (-0.23 to 2.85) | 0.09 |
| 3-year | 80.0 (76.6 to 83.4) | 80.7 (77.3 to 84.1) | 82.0 (78.6 to 85.5) |  |  |  |  |
| 3-year change | -3.38 (-4.52 to -2.25) | -1.73 (-2.86 to-0.60) | -1.63 (-2.74 to -0.53) | **1.65 (0.05 to 3.25)** | **0.04** | **1.75 (0.16 to 3.34)** | **0.03** |
| **Water from all fluids, mL/d** | **1204 ± 250** | **1775 ± 137** | **2409 ± 268** |  |  |  |  |
| N | 303 | 303 | 303 |  |  |  |  |
| **eGFR (ml/min/1.72m^2^)** |  |  |  |  |  |  |  |
| Baseline | 83.2 (79.8 to 86.5) | 83.5 (80.2 to 86.9) | 82.6 (79.2 to 86.0) |  |  |  |  |
| 1-year | 82.1 (78.7 to 85.5) | 83.7 (80.3 to 87.0) | 82.4 (79.1 to 85.8) |  |  |  |  |
| 1-year change | -1.23 (-2.32 to -0.13) | -0.08 (-1.16 to 1.00) | 0.38 (-0.70 to 1.46) | 1.14 (-0.39 to 2.69) | 0.14 | **1.61 (0.07 to 3.15)** | **0.04** |
| 3-year | 80.3 (76.9 to 83.7) | 81.3 (77.9 to 94.7) | 81.1 (77.8 to 84.5) |  |  |  |  |
| 3-year change | -2.89 (-4.01 to -1.76) | -2.38 (-3.50 to -1.26) | -1.45 (-2.57 to -0.33) | 0.50 (-1.08 to 2.09) | 0.53 | 1.43 (-0.15 to 3.02) | 0.07 |
| **Control group (n = 923)** | | | | | | | |
| **Tertiles of water consumption** | | | | | | | |
|  | **T1 (lowest)** | **T2** | **T3 (highest)** | **T2 vs. T1 difference** | **p-value** | **T3 vs. T1 difference** | **p-value** |
| **Total water intake, mL/d** | **2199 267** | **2821 162** | **3531 369** |  |  |  |  |
| N | 308 | 308 | 307 |  |  |  |  |
| **eGFR (ml/min/1.72m^2^)** |  |  |  |  |  |  |  |
| Baseline | 84.8 (82.2 to 87.5) | 82.7 (80.1 to 85.3) | 81.5 (78.8 to 84.1) |  |  |  |  |
| 1-year | 83.4 (80.7 to 86.1) | 80.4 (77.8 to 83.1) | 80.0 (77.3 to 82.6) |  |  |  |  |
| 1-year change | -1.39 (-2.42 to -0.36) | -2.24 (-3.28 to -1.21) | -1.28 (-2.30 to -0.26) | -0.85 (-2.31 to 0.61) | 0.25 | 0.11 (-1.34 to 1.56) | 0.88 |
| 3-years | 81.3 (78.6 to 84.0) | 78.9 (76.3 to 81.6) | 78.8 (76.2 to 81.5) |  |  |  |  |
| 3-years change | -3.74 (-4.79 to -2.69) | -3.83 (-4.89 to -2.77) | -2.18 (-3.23 to -1.13) | -0.09 (-1.58 to 1.39) | 0.90 | **1.55 (0.07 to 3.04)** | **0.03** |
| **Water from all fluids, mL/d** | **1264 ± 236** | **1800 ± 138** | **2395 ± 286** |  |  |  |  |
| N | 308 | 308 | 307 |  |  |  |  |
| **eGFR (ml/min/1.72m^2^)** |  |  |  |  |  |  |  |
| Baseline | 84.9 (82.2 to 87.6) | 82.9 (80.2 to 85.6) | 81.2 (78.5 to 83.8) |  |  |  |  |
| 1-year | 83.2 (80.5 to 85.8) | 80.8 (78.1 to 83.5) | 79.7 (77.0 to 82.4) |  |  |  |  |
| 1-year change | -1.84 (-2.81 to -0.82) | -1.80 (-2.84 to -0.77) | -1.28 (-2.30 to -0.25) | 0.03 (-1.42 to 1.49) | 0.96 | 0.56 (-0.88 to 2.01) | 0.44 |
| 3-years | 81.3 (78.6 to 894.0) | 78.8 (76.0 to 81.5) | 78.9 (76.2 to 81.6) |  |  |  |  |
| 3-years change | -3.89 (-4.93 to -2.85) | -3.95 (-5.01 to -2.88) | -1.91 (-2.96 to -0.86) | -0.05 (-1.54 to 1.43) | 0.94 | 1.97 (0.49 to 3.46) | < 0.01 |
| Linear Mixed Models (β-coefficients (ml/min/1.73m2) and 95% CI) were used to assess the longitudinal associations between types of water consumption at baseline and changes in eGFR (creatinine + cystatin) over 3 years of follow-up. Model was adjusted for age, sex, visit-time, body mass index (kg/m^2^), educational level (primary or lower, secondary or academic or graduate), smoking habit (never, former or current), total energy intake (kcal/day), physical activity (METs min/week in tertiles), prevalence of diabetes (yes/no), hypertension (yes/no), renal drugs use (yes/no), diuretics use (yes/no), energy reduced Mediterranean diet adherence (in tertiles), dietary intakes of total protein(gr/d) and sodium (mg/d), and participating center (in quartiles by number of participants).  Water from all fluids was adjusted for other water source.  Abbreviations: eGFR, estimated glomerular filtration rate. | | | | | | | |
